# Pain Reduction and Financial Incentives to Improve Glucose Monitoring Adherence in a Community Health Center

**DOI:** 10.1371/journal.pone.0114875

**Published:** 2014-12-08

**Authors:** Mary Ann H. Huntsman, Faith J. Olivares, Christina P. Tran, John Billimek, Elliot E. Hui

**Affiliations:** 1 Share Our Selves Community Health Center, Costa Mesa, CA, United States of America; 2 Health Policy Research Institute and Division of General Internal Medicine, University of California Irvine, Irvine, CA, United States of America; 3 Department of Biomedical Engineering, University of California Irvine, Irvine, CA, United States of America; Mayo Clinic, United States of America

## Abstract

Self-monitoring of blood glucose is a critical component of diabetes management. However, patients often do not maintain the testing schedule recommended by their healthcare provider. Many barriers to testing have been cited, including cost and pain. We present a small pilot study to explore whether the use of financial incentives and pain-free lancets could improve adherence to glucose testing in a community health center patient population consisting largely of non-English speaking ethnic minorities with low health literacy. The proportion of patients lost to follow-up was 17%, suggesting that a larger scale study is feasible in this type of setting, but we found no preliminary evidence suggesting a positive effect on adherence by either financial incentives or pain-free lancets. Results from this pilot study will guide the design of larger-scale studies to evaluate approaches to overcome the variety of barriers to glucose testing that are present in disadvantaged patient populations.

## Introduction

Diabetes is a chronic disease requiring continuous medical care and self-management skills to prevent both acute and long term complications [1]. These complications can be devastating to the individual and costly to the health care system. Regular monitoring of blood glucose levels is a critical component of diabetes management, providing direct feedback to both patients and clinicians regarding the effects of health choices and drug therapies on glycemic control. Self-monitoring of blood glucose (SMBG) is a simple procedure that can be performed by a patient outside of the clinical setting. Despite the importance and accessibility of SMBG, adherence to prescribed testing regimens is poor for many patients [2], especially among ethnic minority populations, and patients with language barriers or lower levels of education [3].

A number of barriers to SMBG adherence, including demographic, biological, and psychosocial factors, have been described [2]. Among the psychosocial impediments, environmental factors such as inconvenience, cost, and pain have been reported to be the most important [2], [4], [5]. Given that poor adherence to diabetes self-management regimens among disadvantaged populations cannot be explained entirely by a lack of ability to pay [6], efforts to improve adherence to SMBG solely by reducing out of pocket costs are unlikely to be successful if other important barriers are not addressed.

Of the many barriers to SMBG, pain is one impediment that can be readily addressed by technology. Each blood glucose measurement typically requires a painful finger prick to extract a drop of blood. Recent advances in glucose meters have significantly reduced the required blood volume, enabling new lancet devices that combine a small needle diameter with minimal skin puncture to achieve virtually pain-free blood extraction [7], [8]. Such lancets could reduce a key deterrent to SMBG and result in improved adherence. However, studies to track the effect of these devices on SMBG adherence have not been reported.

Financial incentives have shown promise for encouraging patient compliance in health care. Rewards, typically in the range of $5 to $100, have been found to be effective in encouraging medical and dental visits, immunization, treatment of tuberculosis, and control of weight and blood pressure [9]. Importantly, a recent study of diabetic patients found that financial incentives could have a small positive effect on overall glucose control [10]. Adherence to SMBG was not considered specifically, however.

Although there is evidence suggesting that adherence to SMBG is particularly challenging in disadvantaged populations [3], and that interventions employing pain-reduction and financial incentives may help to lower some of the barriers to adherence [9], [11], studies evaluating the effectiveness of such interventions in low-income community settings can be challenging to conduct due to disparities in education, health insurance, language, income and health status [12]. We present results from a small pilot study (1) to describe the level of SMBG adherence in a low-income community health clinic population consisting largely of non-English speaking ethnic minorities with low health literacy, (2) to assess the feasibility of conducting a larger-scale study to evaluate the effectiveness of interventions to improve adherence, and (3) to examine preliminary evidence for the effectiveness of two interventions to increase SMBG adherence.

## Methods

### Ethics Statement

Prior to subject enrollment, this study protocol was approved by the University of California Irvine Institutional Review Board (UCI IRB HS# 2012-8765). Oral consent was approved by the UCI IRB and chosen on the advice of the clinic personnel, who indicated that some members of their patient population could be uncomfortable with signing a written document. Oral consent was documented as a note in the patient's medical record.

### Setting and Participants

The study setting was a community health clinic located in the city of Costa Mesa in Orange County, CA. Share Our Selves (SOS) serves a population of approximately 7,200 unduplicated patients. 93% of these patients earn an income at or below 100% of the Federal Poverty Level. 59% of the patient population is Hispanic/Latino. 45% of these Hispanic/Latino patients report Spanish as their preferred language. Subjects for the study were drawn from a pool of 150 insulin-dependent diabetics that receive full-scope primary-care services at SOS. The gender ratio of this pool was close to an equal distribution of male and female, and the age range was 30 to 75 years old.

### Study Design, Randomization and Interventions

This pilot study was a randomized controlled trial with three arms. Participants were randomly allocated to one of three groups:

- Control Group: Subjects were provided with a TRUEtrack glucometer, Accu-Chek Safe-T-Pro Plus lancets, and 100 test strips. This system utilizes a standard lancet that is not considered pain-free. No financial incentive was offered.
- Financial Incentive Group: Subjects were provided with a TRUEtrack glucometer, Accu-Chek Safe-T-Pro Plus lancets, and 100 test strips. This system utilizes a standard lancet that is not considered pain-free. Subjects were promised a $50 gift card for successful completion of all assigned readings. They were also informed that fewer completed readings would earn reduced rewards, but the exact payment formula was not discussed.
- Pain-Free Group: Subjects were provided with a TRUEtrack glucometer, Accu-Chek Softclix Plus lancets, and 100 test strips. This system utilizes a pain-free lancet device that testing has shown to provide a substantially more comfortable glucose measurement experience [7], [8]. No financial incentive was provided to this group.

### Outcomes

The primary study endpoint was SMBG adherence, defined as the proportion of prescribed SMBG readings completed by the patient during a 30-day observation period. Loss to follow-up was evaluated as a secondary endpoint as part of the feasibility assessment.

### Sample Size

For this pilot study, we sought to enroll 60 participants, 20 in each study arm, which, allowing for up to 25% loss to follow-up in each arm, would allow us to detect a one standard deviation difference in the primary endpoint between the control group and either intervention group (assuming 1-β = 0.80 and α = 0.05).

### Study Procedure

During the course of normal medical visits, qualified patients were informed about the study and invited to participate. Qualified patients met the following inclusion criteria: (1) greater or equal to 18 years of age, (2) diagnosed with type 1 or type 2 diabetes, (3) limited financial access to a glucose monitoring system (glucometer, lancets, or test strips), and (4) received services at the clinic between July and October 2013. A study team member met with interested patients in order to obtain oral consent, to provide a study information sheet in English or Spanish, and to provide training and instructions. A block randomization schedule (block size  = 6) was employed to assign subjects into three equally sized groups as described above. A total of 60 subjects were enrolled into the pilot study, with 20 subjects in each group. The first 60 patients who met the study criteria were enrolled.

Subjects were instructed to measure their blood glucose levels twice per day over the course of 30 days. After 30 days, subjects returned to the clinic with their glucometer, which stored a record of all glucose measurements. The total number of glucose measurements performed over 30 days was then extracted from the devices and tabulated. For the purposes of this study, no other information was recorded, including demographic data. If subjects recorded more than two measurements on a single day, only two measurements were counted towards their tally. Glucometers, lancets and test strips were provided free of charge to all subjects in order to remove cost as a barrier to testing. Subjects in the financial incentive group were rewarded with gift cards according to the number of measurements that they performed. 50 measurements or more earned a $50 card, 40 measurements or more earned a $40 card, and so forth.

### Data Analysis

Data were analyzed using SPSS v.21 (IBM Corp, Armonk, NY). The shape of the distribution of adherence rates was examined with descriptive statistics, histogram, quantile-quantile plots and the Lilliefors corrected Kolmogorov-Smirnov test [13]. Differences between group means were assessed with independent samples t-tests. Loss to follow-up rates were compared using chi-squared tests.

## Results

A total of 60 subjects were enrolled in the study, with 20 subjects assigned to each group. 10 out of the 60 subjects (17%) were lost to follow-up and did not return to present their glucometer for data collection. 3 subjects (15%) in the control group, 1 subject (5%) in the financial incentive group, and 6 subjects (30%) in the pain-free group were lost to follow-up. Group differences in rates of loss to follow-up were not statistically significant (p = 0.10), but suggest a trend of differential attrition rates that may warrant further investigation.

**Figure 1:**
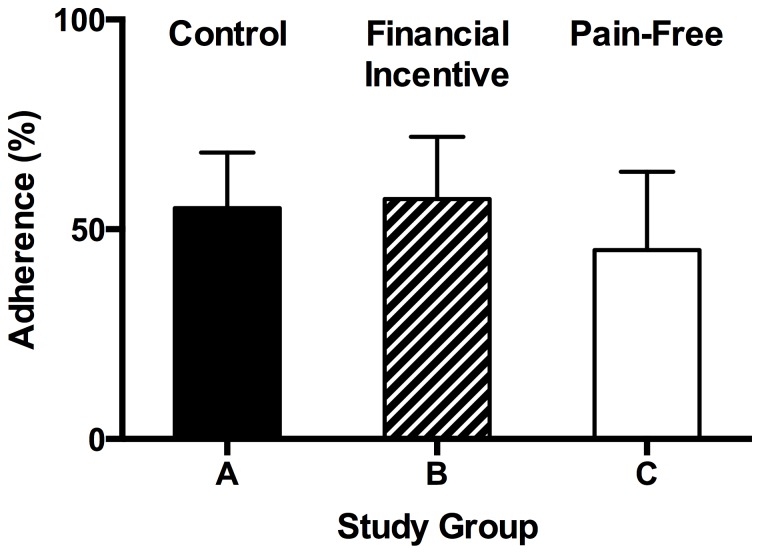
Mean adherence was uniform across study groups. Mean adherence in the control, financial incentive, and pain-free groups did not differ by a significant margin. Error bars represent 95% confidence intervals.

Subjects who completed the study (n = 50) had mean SMBG adherence rates of 53% (SD = 29%). The distribution of SMBG adherence rates approximated the normal distribution (Lilliefors corrected Kolmogorov-Smirnov p = 0.2). Compared to the control group (mean [SD] SMBG adherence = 55% [26%]), SMBG adherence was similar in the financial incentive group (mean [SD] = 57% [31%]; t(34) = −0.24, p = 0.81) and in the pain free group (mean [SD] = 45% [32%]; t(29) = 0.94, p = 0.36; see Fig. 1). Across all groups, 22% of all subjects achieved at least 80% adherence.

## Discussion

Results from this pilot study suggest that it is feasible to study the effectiveness of interventions employing financial incentives and pain-free lancets on SMBG adherence in a community health clinic serving disadvantaged patients with diabetes. We observed that patients were receptive to participating in studies both for a “positive” intervention (providing a financial incentive in addition to covering the cost of testing supplies) and for a “negative” intervention (reducing the pain of finger sticks for SMBG). Further, patients who returned for 30-day follow-up reported little trouble using the SMBG testing equipment, and clinic personnel could be trained to download SMBG results and complete the study protocol.

Across all three study groups, SMBG adherence rates were low, but similar to previously reported findings [2], underscoring that non-adherence to SMBG is a widespread issue in diabetes care. Given the limitations of this pilot study, including small sample size and missing data, it is not possible to conclude whether or not the specific interventions that we examined can improve SMBG adherence. Although the study produced no preliminary evidence for the effectiveness of either intervention approach compared to the control condition, we determined that providing no-cost testing supplies to participants in all three conditions was adequate to attain our recruitment goal in a short period of time and to retain a large proportion of patients at 30-day follow-up.

### Lessons For Future Research

Future, larger-scaled studies should be designed with several considerations in mind. First, SMBG adherence rates were highly variable, with standard deviations approaching 30% (more than half the mean SMBG adherence rate). Sample size calculations should account for a high relative standard deviation to ensure adequate statistical power.

Second, although loss to follow-up rates were similar across the three groups, there was a non-significant trend suggesting that loss to follow-up may be more common in the pain-free intervention group than the other groups. Anecdotally, one subject complained that the pain-free lancets were more difficult to use in comparison to standard lancets. For each measurement, the pain-free lancet system requires a needle cartridge to be loaded into a spring actuator unit that fires the needle with a tunable and repeatable force. In contrast, the standard lancet simply needs to be uncapped and pressed into the skin. It is possible that the complexity of the pain-free lancing system is related to the high number of subjects in the pain-free group who dropped out of the study. Future studies should be designed to account for at least 17% loss to follow-up, and possibly as high as 30% if pain-free lancets are to be employed. Additional participant incentives or more regular contact with participants may help reduce loss to follow-up further. Finally, if investigators wish to address missing data using multiple imputation or random effects models, they must design the study to include an adequate number of observations and adequate data on participants to employ those approaches [14].

Third, given the lack of evidence for an effect of the financial incentive intervention, alternative incentive protocols should be considered to maximize impact. Patients may be more strongly incentivized if they are allowed to chose for themselves which reward they will be working to earn [15]. The strength of an incentive may be influenced by individual preference, age, financial status, or cognitive abilities and thus weighted differently by each member of the randomly assigned incentive group [16].

## Conclusions

Adherence to self-monitoring of blood glucose is a multifaceted challenge that may require complex approaches to address effectively. While pain and cost have been identified as important barriers to SMBG, our pilot study did not demonstrate a significant benefit towards overcoming these barriers by providing pain-free lancets or financial incentives. However, conducting such a study in a resource-limited community health center population is feasible. Larger scale studies powered to account for loss to follow-up and the relatively high variability in SMBG will allow researchers to test various approaches to help low-income patients overcome barriers to SMBG.
